# Supplementary material for: Should homes and workplaces purchase portable air filters to reduce the transmission of SARS-CoV-2 and other respiratory infections? A systematic review
Source: PLoS One. 2021 Apr 29;16(4):e0251049. doi: 10.1371/journal.pone.0251049 (PMC8084223; doi:10.1371/journal.pone.0251049)
Supplement: S1 Table — (DOCX) [file pone.0251049.s001.docx]

**S1 Table. Medline Search Strategy**

| 1 | Exp Air Filters |
| --- | --- |
| 2 | air adj3 (filter* or filtration or cleaning or cleaner* or purification or purifier* or respirator*).tw |
| 3 | HEPA filter.tw |
| 4 | HEPA.mp |
| 5 | CADR.tw |
| 6 | 1 or 2 or 3 or 4 or 5 |
| 7 | Exp Infections |
| 8 | Exp Infection Control |
| 9 | Exp Viruses |
| 10 | Exp Bacteria |
| 11 | Exp Air Microbiology |
| 12 | Exp Cross Infection |
| 13 | (infection* or bacteria or virus*).tw |
| 14 | air microbiology.tw |
| 15 | 7 or 8 or 9 or 10 or 11 or 12 or 13 or 14 |
| 16 | 6 and 15 (15,750) |
