# Supplementary material for: Should homes and workplaces purchase portable air filters to reduce the transmission of SARS-CoV-2 and other respiratory infections? A systematic review
Source: PLoS One. 2021 Apr 29;16(4):e0251049. doi: 10.1371/journal.pone.0251049 (PMC8084223; doi:10.1371/journal.pone.0251049)
Supplement: S2 Table — (DOCX) [file pone.0251049.s002.docx]

**S2 Table.** **Summary of studies based on eligibility criteria and author best judgement (non-exhaustive list)**

| **No.**  **(DOI)** | **Author (publication year) [reference no.]** | **Description** | **Country** | **Setting** | **Type of filter** | **Portable (Y/N)** | **Include in review (Y/N)** | **Reason for exclusion** |
| --- | --- | --- | --- | --- | --- | --- | --- | --- |
| 1  ([10.1038/s41598-020-63543-1](https://www.ncbi.nlm.nih.gov/pmc/articles/PMC7156680/pdf/41598_2020_Article_63543.pdf)) | Guo et al (2020) [15] | This study collected samples from portable HEPA air purifiers and floor dust inside 12 offices over a 12-month period. They investigated the characteristics of the  bacterial communities, bacterial diversity and bacterial sources in the HEPA filters compared to floor dust samples. | China | Offices | HEPA | Y | Y | n/a |
| 23  ([10.1016/j.buildenv.2020](https://www.ncbi.nlm.nih.gov/pmc/articles/PMC7424318/pdf/main.pdf)) | Mousavi et al (2020) | Aerosols were released into a specially designed patient isolation room with a temporary anteroom with plastic barrier, beside the space at the patient bed where the patient’s head would be located, to simulate the spread of SARS-CoV-2 virus from an infected patient. Portable HEPA filters were placed at the bottom of the patient bed and outside of the plastic barrier. | USA | Patient isolation room in a healthcare facility | HEPA (Abatement Technologies® PAS2400) | Y | N | The patient isolation room was specially designed as part of the experiment. The aerosols released into the room were not SARS-CoV-2 but particles of different sizes. |
| 3  ([10.1016/j.ajic.2018.12.019](https://www.ajicjournal.org/article/S0196-6553(18)31182-9/fulltext)) | Bischoff et al (2019) [16] | Air sampling was performed for 20 mins at the head and foot of patients bed in an emergency room setting, and at the doorway exit or entrance of the room at baseline, and then again after the HUAIRS filtration system was run for 8 air exchanges. Samples were collected on blood agar plates that were then incubated for 48 hours before counting bacterial growth as colony forming units. | USA | Emergency rooms | HUAIRS (Combination of HEPA filtration, photochemical oxidation, and germicidal UV irradiation) | Y | Y | n/a |
| 4  ([10.1016/j.envres.2019.108749](https://reader.elsevier.com/reader/sd/pii/S0013935119305468?token=33CDE0A335655D29FD9104C7B161C3328CFC257D38D42239BBFED88032E80EE61DDB40D26AAD7965AFA1A3B964C61CE8)) | Gao et al (2019) | The study evaluates the short-term effects of a filtered fresh air ventilation system on classroom indoor air and biomarkers in saliva and nasal samples in preschool children. Two classrooms (n = 43 children) were selected and fresh air ventilation systems (FAVS) with HEPA were installed. | China | Classrooms | HEPA | N | N | Non-portable filter. |
| 5  ([10.1016/j.ajic.2019.03.026](https://www.ajicjournal.org/article/S0196-6553(19)30209-3/fulltext)) | Hakim et al (2019) | A pre- and post-intervention study which compared six test locations, where continuous shielded UV-C air disinfection devices were installed, with 10 control locations without UV-C. Pre- and post-intervention air and surface samples were collected for bacterial and fungal cultures. | USA | Paediatric oncology outpatient unit | Ultraviolet-C disinfection devices | N | N | Non-portable filter. |
| 6  ([10.1007/s10661-019-7876-3](https://link.springer.com/content/pdf/10.1007/s10661-019-7876-3.pdf)) | Lee et al (2019) | The bacterial strain *S. epidermidis* was injected using a nebulizer into a test chamber, which was designed to be similar to a consumer living space (60 m^3^). Microbial sampling was conducted from the HEPA filter inside the test chamber, and the reduction in *S. epidermidis* growth was monitored by performing three consecutive tests. | Korea | Test chamber | HEPA | NR | N | Conducted within a germ-free chamber. |
| 7  ([10.1111/ina.12495](https://onlinelibrary.wiley.com/doi/epdf/10.1111/ina.12495)) | Gologit-Szymczak et al (2018) | The study was designed to assess employees exposure to bacterial and fungal aerosols in offices, including the influence of mechanical ventilation systems. | Poland | Offices | NR (air conditioning and mechanical supply-exhaust ventilation) | N | N | Non-portable filter, used air conditioning and central mechanical systems |
| 8  ([10.1111/ina.12202](https://onlinelibrary.wiley.com/doi/epdf/10.1111/ina.12202)) | Kolarik et al (2016) | The study investigated whether there is an association between ventilation in day care centres and sick leave among nursery children. | Denmark | Day care centres | NR (mechanical or natural ventilation measured) | N | N | Non-portable filter, mainly measured natural ventilation. |
| 9  (<https://pubmed.ncbi.nlm.nih.gov/26528369/>) | Jafari et al (2015) | The study determined the effect of ventilation system parameters and patient bed arrangements on the concentration of airborne pathogens in indoor air of an isolation room. | Iran | Single-bed hospital room | NR (wall or ceiling mounted ventilation systems) | N | N | Non-portable filter. |
| 10  ([10.1111/j.1600-0668.2012.00787.x](https://www.ncbi.nlm.nih.gov/pmc/articles/PMC7201892/pdf/INA-23-50.pdf)) | Korves et al (2013) | Samples from 61 aircraft high-  eﬃciency parti culate air (HEPA) ﬁlters were analyzed with a custom microarray  of 16S rRNA gene sequences (PhyloChip), representing bacterial lineages  Samples from 61 aircraft high-  eﬃciency parti culate air (HEPA) ﬁlters were analyzed with a custom microarray  of 16S rRNA gene sequences (PhyloChip), representing bacterial lineages  Samples from 61 HEPA filters were collected and analysed using a custom microarray of 16S rRNA gene sequences (PhyloChip). | USA | Aircrafts | HEPA | N | N | Aircraft study, non-portable filter. |
| 11  ([10.1111/jocn.12286](https://onlinelibrary.wiley.com/doi/epdf/10.1111/jocn.12286)) | Vokurka et al (2013) | The study investigated the impact on incidence of pneumonia and mortality after haematopoietic stem cell transplantation (HSCT) in HEPA-filtered and non-HEPA-filtered rooms. | Czech Republic | Patient hospital rooms | HEPA | N | N | Non-portable filter. |
| 12  ([10.1016/j.ajic.2010.10.036](https://www.ajicjournal.org/action/showPdf?pii=S0196-6553%2811%2900086-1)) | Goyal et al (2011) | HVAC filters from two large public buildings in Minneapolis and Seattle were sampled to determine the presence of human respiratory viruses and viruses with bioterrorism potential. | USA | Public buildings | HVAC (dust filters installed in heating, ventilation and air conditioning systems) | N | N | Non-portable filter. |
| 13  ([10.1016/j.jaerosci.2008.09.003](https://reader.elsevier.com/reader/sd/pii/S0021850208001729?token=4132224B001A98224B0607C034E10109748C4F80414CE6089E2F18220B26B7EA0682D280B0283F0DAEFC67408CA4EA98)) | Li et al (2009) | The study compared an experimentally designed alumina nanofiber filter with three commercially available HEPA filters to evaluate the performance of the alumina nanofilter for removal and retention of viral aerosols. | USA | Test chamber | HEPA, plus an experimental nanofiber filter | NR | N | Conducted within a germ-free chamber. |
| 14  ([10.1111/j.1365-2672.2007.03569.x](https://sfamjournals.onlinelibrary.wiley.com/doi/epdf/10.1111/j.1365-2672.2007.03569.x)) | Luna et al (2007) | The study evaluated the UCV/HEPA filter’s ability to inactivate *Bacillus atropheus* and *B. anthracis* spores. | USA | Biosafety lab | UVC/HEPA | N | N | Non-portable filter. |
| 15  (<https://www.ncbi.nlm.nih.gov/pmc/articles/PMC1477932/>) | Dee et al (2006) | The authors compared four methods for the reduction of aerosol transmission of Porcine reproductive and respiratory syndrome virus (PRRSV): high-efficiency particulate air (HEPA) filtration, low-cost filtration, bag filtration, and use of a filter tested against particles derived from dioctylphthalate. | USA | Chamber within an experimental animal facility | Multiple filters used, including HEPA (no further details provided other than it was a model of a commercially available filter) | NR | N | Conducted within a germ-free chamber. |
| 16  ([10.1080/10473289.2005.10464612](https://pubmed.ncbi.nlm.nih.gov/15796111/)) | Kujundzic et al (2005) | The study assessed the effectiveness of a new generation of high-volume, ceiling-mounted HEPA-ultraviolet (UV) air filters (HUVAFs) for their ability to remove or inactivate bacterial aerosols. | USA | Indoor therapy pool | Hybrid HEPA-UV air filters known as HUVAFs | N | N | Non-portable filter. |
| 17  ([10.1016/j.annemergmed.2004.07.451](https://www.annemergmed.com/action/showPdf?pii=S0196-0644%2804%2901210-7)) | Mead et al (2004) | The study evaluated the potential feasibility of expedient, negative-pressure HEPA–filtered patient enclosures for control of airborne pathogens during emergencies requiring isolation surge capacity. | USA | Patient hospital rooms | HEPA | Y | N | Study replicates airbourne pathogens; our review is interested in studies that measure airborne pathogens. |
| 18  ([10.1046/j.1442-200x.2003.01746.x](https://onlinelibrary.wiley.com/doi/epdf/10.1046/j.1442-200X.2003.01746.x)) | Suzuki et al (2003) | The authors examined the spread of  Varicalla Zoster Virus (VZV) within the household environment from patients with (VSV) who started oral acyclovir within 24 h after onset of the disease. Swab samples from the  throats of the patients and family members, and from the filters of commercially available air purifiers placed in their houses were collected frequently during the initial seven days after onset of the disease. | Japan | Households | NR (states commercially available air filters used) | NR | N | Study conducted in 1998 (pre-2000). |
