# Supplementary material for: Should homes and workplaces purchase portable air filters to reduce the transmission of SARS-CoV-2 and other respiratory infections? A systematic review
Source: PLoS One. 2021 Apr 29;16(4):e0251049. doi: 10.1371/journal.pone.0251049 (PMC8084223; doi:10.1371/journal.pone.0251049)
Supplement: Attachment — Submitted filename: Response to reviewers - PONE-D-20-34977R1.docx [file pone.0251049.s004.docx]

**Should homes and workplaces purchase portable air filters to reduce the transmission of SARS-CoV-2 and other respiratory infections? A systematic review (PONE-D-20-34977)**

| **No.** | **Reviewer comments** | **Author response** | **Manuscript changes** |
| --- | --- | --- | --- |
|  | **Editor comments** | | |
| 1. | After reading the manuscript, I suggest to increase the complexity of the presentation by shifting tables S1 and S3 and figure S1 in the main text. Furthermore, you need to provide a table presenting the summary of selected studies (n= 18) based on eligibility criteria and best judgment and point out that it could be a non-exhaustive list since a degree of subjectivity may be present (see an example in supplementary of <https://www.mdpi.com/2073-4433/9/4/150/htm> - table S2). A better clarification of the database selection should be also included (one may ask why Clarivate WoS was not consulted?). | We thank the editor for their comments. We have taken the advice of the editor and included Table S1 and S3 and Figure S1 in the main manuscript. We have also added a table (S2) summarising the 18 studies we screened for eligibility. Please accept our apologies for any confusion caused regarding the search engines used, this was an error on our part, we have corrected this as Web of Science was used as part of our search. Please also note that we have updated our search to include papers up to March 2021, but found no additional studies which were eligible. | Line 87, Table 1 (formerly S1) added.  Line 126, Table 2 (formerly S3) added.  Line 178, Fig. 2 (formerly S1 Fig) added.  Line 98, page 3 (typo corrected).  Line 94, updated search dates. |
| 2. | In your Data Availability statement, you have not specified where the minimal data set underlying the results described in your manuscript can be found. PLOS defines a study's minimal data set as the underlying data used to reach the conclusions drawn in the manuscript and any additional data required to replicate the reported study findings in their entirety. All PLOS journals require that the minimal data set be made fully available. For more information about our data policy, please see <http://journals.plos.org/plosone/s/data-availability>. | As this is a systematic review, we did not generate a dataset as such. All papers screened are published papers. We have included our full search strategy in S1 Table which should ensure readers could reproduce our findings, plus a summary of our eligibility screening of our final 18 full text papers screened (S2). We have no other data which could be provided which would enable readers to reproduce our findings, though are very happy to discuss if necessary. | No changes. |
|  | **Reviewer 1 comments** | | |
| 3. | Limited number of studies. Based on authors’ search strategy, authors did not found any study investigating the effects of portable, commercially available air filters on incidence of respiratory infections. This review only found two papers which investigated whether portable air filters can reduce airborne bacteria from the air. These very limited number of studies reduce the significance of this manuscript. I would recommend authors to expand research criteria (such as including centralized HVAC system with air filter studies etc.) to include more studies. Expanding beyond portable air filter studies would provide important evidence of the efficacy of using other air filtration technology in reducing transmission of COVID-19. | Whilst we agree that this review found only two papers which were eligible, we do not feel that this reduces the overall significance of the manuscript. Our review was exploring portable air filtration as it is an easily-implementable, relatively affordable ‘real-world’ intervention that can be easily fitted into most indoor settings to try to minimise transmission of infections including SARS-CoV-2. We explored the possibility of including non-portable filters in this review, including HVAC, and those used in operating theatres and aircraft, however we believe this warrants a separate review. Although this of course would provide important evidence as to the usefulness of non-portable filters in reducing respiratory infection, they are not as easily implemented as portable filters, and do not therefore necessarily provide a ‘real-world’ solution which members of the public could easily adopt if it was proven to be effective. In addition, including both portable and non-portable air filter studies would have added considerable time onto our data extraction process (we estimate at least 40 additional studies) and we feel that this review is timely and should be published as soon as possible, with a further review to follow (which we are currently working on) exploring the effectiveness of non-portable air filter systems in preventing infections. | No changes. |
| 4. | The two studies included in the systematic review only focused on air filter use on bacterial in the air, but not virus. Given the different size and pathological characteristics between bacteria and virus, I would encourage authors add some more discussions on how the results from bacteria can inform controlling virus. | We have added some additional discussion around this as the reviewer suggested. | Additional text added to line 287-294. |
| 5. | In this manuscript, there is only 1 figure, but no tables. I would like encourage authors to include 1-2 tables summarizing two studies they included in the synthesis. | We have added a study eligibility table as a supplementary table (S2) which includes details on the two studies included in the paper, as well as the additional 16 papers we screened but concluded were not eligible. We have also included some of our previous supplementary tables and figure in the main text. | Line 87, Table 1 (formerly S1) added.  Line 126, Table 2 (formerly S3) added.  Line 178, Fig. 2 (formerly S1 Fig) added. |
|  | **Reviewer 2 comments** | | |
| 6. | The included two studies are largely different in indoor settings (office vs. emergency room), sampling duration (one year vs. one day?), air filter type (air purifier with HEPA filter vs. high-efficiency particulate air-ultraviolet air recirculation system). Additionally, the missing information (e.g., filter specifications, filter use duration/frequency as well as the air exchange of the indoor environment etc.) makes the author’s claim on the ability of air filters to capture airborne bacteria less convincing. The author may want to consider expanding the scope of this review to also include non-portable HEPA filters. As the author recognized, the within-building filtered air flow systems such as HVAC systems are commonly equipped in office and domestic settings in UK and worldwide. It is certainly an important ‘real-world’ setting as well. Including this body of literature may provide additional information and evidence on the effectiveness of air filters in reducing respiratory infections or capturing airborne bacteria or viruses. It may also help answer other practical questions such as should we recommend people who stay in the indoor environment with within-building air filtration to acquire additional portable air filters? | Please see above response (3) to reviewer 1. | No changes. |
| 7. | I really like that the author also summarized literatures that do not fit into the review scope/inclusion criteria but relevant to the topic in the discussion section. It provides a great overview of the existing literature on this topic. But for the discussion on future research, I would like to see more details rather than an oversimplified statement that randomized controlled trials are urgently needed. What are the research questions need to be answered? What evidences are needed to make concrete recommendations and what are the immediate challenges to overcome? | We have added in some additional text to our discussion section as the reviewer has suggested. | Additional text added to line 353-355. |
| 8. | Line 59 – 62: References? | We have added a reference to line 62. | Line 62, reference added. |
| 9. | Line 64: References? | We have added a reference to line 64. | Line 64, reference added. |
| 10. | Line 67 – 69: Not clear why this study is cited here. It is not related to air filters as the sentence before and after. Perhaps a reference to the statement in line 59 - 62? | On reflection, we agree that this study cited between lines 67-69 (reference 11 in previous manuscript version) is not entirely related to the air filters we describe in the rest of the paragraph. We have removed this sentence as we do not feel it is needed. | Sentence removed from lines 67-69. |
| 11. | The review seems to focus on effectiveness, but the one included study was assessing the efficacy. The authors may want to be clear about the scope and make sure “effectiveness” and “efficacy” are used carefully and consistent throughout the manuscript. | We agree with the reviewer that we could have been more clear around the use of the word efficacy. Upon reading the manuscript again, we have amended the use of ‘efficacy’ to ‘effectiveness’ as this better reflects what we mean. | Line 184, text amended.  Line 239, text amended.  Line 304, text amended. |
