# Supplementary material for: Should homes and workplaces purchase portable air filters to reduce the transmission of SARS-CoV-2 and other respiratory infections? A systematic review
Source: PLoS One. 2021 Apr 29;16(4):e0251049. doi: 10.1371/journal.pone.0251049 (PMC8084223; doi:10.1371/journal.pone.0251049)
Supplement: Attachment — Submitted filename: Response to reviewers.docx [file pone.0251049.s005.docx]

|  |  |  |  | |
| --- | --- | --- | --- | --- |
|  |  | | | |
|  |  | | **centre for ACADEMIC PRIMARY CARE**  NIHR School of Primary Care Research  Bristol Medical School  Population Health Sciences  Canynge Hall, 39 Whatley Road  Bristol, BS8 2PS | |
|  |  |  | Ashley Hammond  Senior Research Associate  ashley.hammond@bristol.ac.uk  15^th^ April 2021 | |
| Dear Dr Dunea, | | | |  |

**Re: Should homes and workplaces purchase portable air filters to reduce the transmission of SARS-CoV-2 and other respiratory infections? A systematic review (PONE-D-20-34977)**

Many thanks for your 14 April 2021 email inviting us to revise our manuscript. As requested, two versions are submitted (‘Revised Manuscript with Track Changes’ and ‘Manuscript’).

The table below describes our responses to editor and reviewer comments, and the changes made to the manuscript where necessary.

Yours sincerely,

Ashley Hammond

**Should homes and workplaces purchase portable air filters to reduce the transmission of SARS-CoV-2 and other respiratory infections? A systematic review (PONE-D-20-34977)**

| **No.** | **Editor/Reviewer comments** | **Author response** | **Manuscript changes** |
| --- | --- | --- | --- |
| 1. | Acknowledge the review's limitations, make Suggestions for Further Research  For these two points consider the comments of both reviewers: - "elaborate more on the future directions of research (e.g. specific indoor settings, population, cost-benefit, uptake/adoption and other practical implications) to better reflect the manuscript title: “Should homes and workplaces purchase portable air filters…”, as well as the focus of effectiveness. " - "citing 2 papers in a review is relatively weak." | We agree that more clarity could be provided around the limitations of the study, and have added some additional detail to our manuscript. We have also added some additional details on future research. | Text added, lines 282-285.  Text added, lines 354-361. |
| 2. | Although your study may offer important insights about the research problem, other questions related to the problem likely remain unanswered. Moreover, some unanswered questions may have become more focused because of your study. You should make suggestions for further research in the discussion section. | We have added additional text to our discussion section regarding directions of future research. | Text added, lines 354-361. |
| 3. | Include a Conclusion section synthesizing the key findings and ideas. | We agree that a conclusion section would be helpful to bring our study findings together, and therefore have added this in to our manuscript. | Text added, lines 363-374. |
